# Alveolar Soft Part Sarcoma of the Uterus: Clinicopathological and Molecular Characteristics

**DOI:** 10.3390/diagnostics12051102

**Published:** 2022-04-27

**Authors:** Yurimi Lee, Kiyong Na, Ha Young Woo, Hyun-Soo Kim

**Affiliations:** 1 Department of Pathology and Translational Genomics, Samsung Medical Center, Sungkyunkwan University School of Medicine, Seoul 06351, Korea; yrm.lee@samsung.com; 2 Department of Pathology, Chungnam National University School of Medicine, Daejeon 35015, Korea; 3 Department of Pathology, Kyung Hee University Hospital, Kyung Hee University College of Medicine, Seoul 02447, Korea; raripapa@gmail.com

**Keywords:** uterus, alveolar soft part sarcoma, immunohistochemistry, next-generation sequencing

## Abstract

Alveolar soft part sarcoma (ASPS) is a rare malignant mesenchymal tumor mainly affecting adolescents and young adults, with a predilection for the deep soft tissues of extremities. ASPS arising in the female genital tract is extremely rare and poses a significant diagnostic challenge. We herein present two rare cases of ASPS, one occurring in the uterine corpus of a 27-year-old woman, and the other in the uterine cervix of a 10-year-old girl. We described the clinical, histological, immunophenotypical, and molecular characteristics of primary uterine ASPS. We performed immunostaining for transcription factor E3 (TFE3), human melanoma black 45 (HMB45), melan-A, desmin, pan-cytokeratin (CK), paired box 8 (PAX8), CD10, hormone receptors, and S100, and targeted RNA and DNA sequencing using commercially available cancer gene panel. In case 1, a 27-year-old woman was referred to our hospital after laparoscopic uterine myomectomy at an outside hospital. Imaging studies revealed a residual tumor in the uterine corpus. In case 2, a 10-year-old girl underwent surgical excision for the cervical mass and was diagnosed as having ASPS. She was then referred to our hospital for further management. Both patients received total hysterectomy. Histologically, they displayed characteristic histological features of ASPS. Strong nuclear TFE3 immunoreactivity, periodic acid-Schiff-positive, diastase-resistant intracytoplasmic rod-shaped crystalloids or granules, and the identification of *ASPSCR1*–*TFE3* fusion confirmed the diagnosis of ASPS in both cases. Lack of immunoreactivity for HMB45, melan-A, desmin, pan-CK, PAX8, and S100 excluded the possibility of perivascular epithelioid cell tumor, clear cell sarcoma, metastatic renal cell carcinoma, granular cell tumor, and paraganglioma. Our observations can help pathologists make an accurate diagnosis of uterine ASPS and suggest that pathologists should include primary uterine ASPS in the differential diagnosis of uterine mesenchymal tumors.

## 1. Introduction

Alveolar soft part sarcoma (ASPS) is a rare malignant mesenchymal tumor that primarily affects adolescents and young adults [1,2]. ASPS comprises less than 1% of all soft tissue sarcoma cases [3]. Although this tumor tends to grow slowly and behave in a clinically indolent fashion, it has a significant risk of metastatic spread, often early in the disease course [3]. Early detection, accurate diagnosis, and appropriate management of ASPS are essential for improving the patient’s chance of survival. Prognostic factors for ASPS include tumor size, age of presentation, and the development of metastasis [4].

The female genital tract is an extremely rare site of origin for ASPS. This report presents two rare cases of ASPS, one occurring in the uterine corpus and the other in the cervix. We herein describe the clinical, histological, immunophenotypical, and molecular characteristics of primary uterine ASPS comprehensively.

## 2. Materials and Methods

### 2.1. Case Selection and Clinicopathological Data Collection

We found two uterine ASPS cases from surgical pathology archives, using the combination of keywords ‘alveolar soft part sarcoma’, ‘uterus’, ‘corpus’, ‘cervix’, ‘vagina’, and ‘vulva’. Clinical information—including age of patient at diagnosis, presenting symptom, magnetic resonance imaging (MRI) finding, positron emission tomography–computed tomography (PET-CT) finding, preoperative clinical impression, surgical procedure, postoperative treatment, postoperative recurrence and metastasis, current status, and disease-free survival period—was obtained from the electronic medical records and pathology reports. A single board-certified gynecological pathologist thoroughly reviewed all available hematoxylin and eosin (H&E)-stained slides using light microscopy. Pathological information—including the location and greatest dimension of tumor, lymphovascular space invasion (LVSI), histological growth pattern, nuclear pleomorphism, mitotic activity, and tumor cell necrosis—was collected. For case 1, the most representative slide was selected to perform immunohistochemical staining and next-generation sequencing (NGS). For case 2, in contrast, the outside unstained slides obtained from the mass excision specimen were used.

### 2.2. Immunohistochemical Staining

Four-micrometer-thick, formalin-fixed, paraffin-embedded (FFPE) slices were deparaffinized and rehydrated using a xylene and alcohol solution. Immunostaining was performed using automated instruments [5,6,7,8,9,10,11,12,13,14,15,16]. After antigen retrieval, the slices were incubated with the primary antibodies listed in Table 1. After chromogenic visualization, the slices were counterstained with hematoxylin. Appropriate positive and negative controls were concurrently stained to validate the staining method. Negative controls were prepared by substituting non-immune serum for primary antibodies, resulting in no detectable staining.

### 2.3. Special Staining

Four-micrometer-thick FFPE slices were stained with the periodic acid-Schiff method with diastase digestion (PAS-D) to examine the presence of coarse cytoplasmic granularity or crystalline inclusions, supporting the diagnosis of ASPS.

### 2.4. Nucleic Acid Extraction

Five-micrometer-thick FFPE slices were deparaffinized and rehydrated using a xylene and alcohol solution. The sections were manually microdissected under a dissecting microscope using a scalpel point dipped in ethanol. The scraped material was washed in phosphate-buffered saline and digested overnight in proteinase K at 56 °C in Buffer ATL (Qiagen, Germantown, CA, USA). DNA and RNA were isolated using the QIAamp DSP DNA FFPE Tissue Kit (Qiagen) [17,18,19,20,21]. A Qubit 4.0 Fluorometer (Thermo Fisher Scientific, Waltham, MA, USA), a highly sensitive and accurate fluorescence-based quantitation assay, was used for sample quantitation.

### 2.5. NGS

NGS library preparation was performed using the extracted DNA and RNA, Ion AmpliSeq Library Preparation (Thermo Fisher Scientific), and IonChef System (Thermo Fisher Scientific) [17,18,19,20,21]. We applied the Oncomine Comprehensive Assay v3 (Thermo Fisher Scientific), which is an amplicon-based targeted assay that enables the detection of relevant single-nucleotide variants, amplifications, gene fusions, and indels from 161 unique genes. Sequencing was performed using the IonTorrent S5 XL platform (Thermo Fisher Scientific) and positive control cell line mixtures (Horizon Discovery, Cambridge, UK). Genomic data were analyzed and alterations were detected using the IonReporter Software 5.6 (Thermo Fisher Scientific). We also manually reviewed the variant call format file and Integrated Genomic Viewer (Broad Institute, Cambridge, MA, USA). Pathogenic variants in coding regions, promoter regions, or splice variants were retained.

## 3. Case Presentation

### 3.1. Case 1: Primary ASPS of the Uterine Corpus

#### 3.1.1. Clinical Presentation

**Figure 1:**
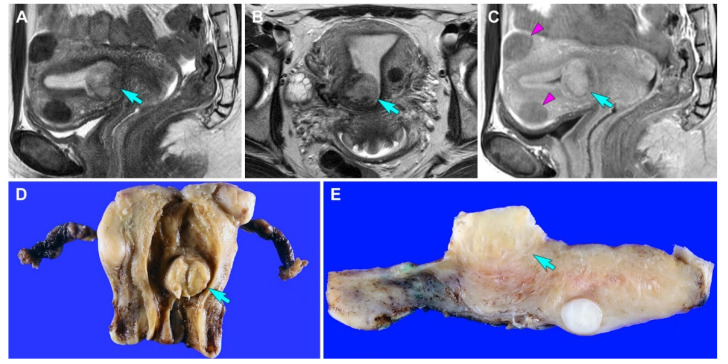
Imaging and gross findings (case 1). (**A**,**B**) T2-weighted (**A**) sagittal and (**B**) axial magnetic resonance imaging (MRI) reveals a relatively well-circumscribed mass in the uterus (blue arrows). (**C**) Contrast-enhanced T1-weighted turbo spin-echo sagittal MRI reveals that the tumor appears to involve the superficial myometrium. The signal intensity of the uterine mass (blue arrow) is higher than that of intramural leiomyomas (purple arrowheads). (**D**) Grossly, a well-circumscribed mass (blue arrow) is located in the uterine corpus. (**E**) The cut section shows an exophytic mass (blue arrow) that appears to invade the superficial myometrium.

A 27-year-old woman presented with vaginal bleeding. She underwent transvaginal ultrasonography at a local clinic, which revealed a 2.5 cm uterine mass. Laparoscopic myomectomy was performed based on the clinical impression of uterine leiomyoma. Her final pathological diagnosis was uterine ASPS. She was then referred to our hospital. Pelvic MRI revealed a suspected residual tumor at the previous myomectomy site (Figure 1A–C). PET-CT revealed increased fluorodeoxyglucose uptake within the uterine corpus. No remarkable uptake was observed elsewhere in the body. She underwent a total hysterectomy.

#### 3.1.2. Pathological Findings

**Figure 2:**
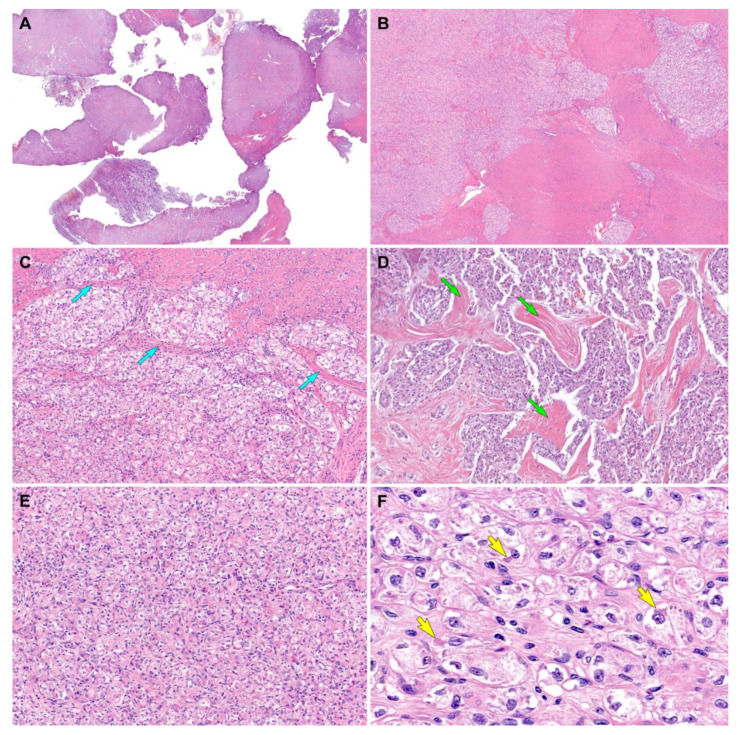
Histological findings (case 1). (**A**) Scanning-power magnification reveals that the myomectomy specimen consists predominantly of tumor tissue infiltrating the myometrium. (**B**) Low-power magnification reveals that the tumor irregularly permeates the myometrium and forms variable-sized islands of tumor cells. (**C**) Thin fibrovascular septa (blue arrows) surround the tumor cell nests. (**D**) The tumor cells were organized in irregular-shaped sheets embedded in a background of dense hyalinized stroma (green arrows). (**E**) A solid, diffuse growth pattern with little or no intervening stroma is also noted. (**F**) The high-power view demonstrates large polygonal cells possessing clear or vacuolated cytoplasm and distinct cell borders. In some microscopic foci, eosinophilic intracytoplasmic materials (yellow arrows) are located closely adjacent to the nuclei. Original magnification: (**A**) 10×; (**B**) 20×; (**C**,**D**) 40×; (**E**) 100×; (**F**) 400×.

Grossly, a 2.2 cm well-circumscribed mass was identified in the lower uterine segment. The cut section showed a yellow-tan, rubbery, exophytic mass that appeared to invade the superficial myometrium (Figure 1D). A few intramural leiomyomatous nodules were also observed in the myometrial outer half (Figure 1E). Histologically, the epicenter of the tumor was located between the myometrium and endometrial stroma. The proliferative endometrial glands were unremarkable. Low-power magnification revealed that the tumor formed variable-sized cellular islands and irregularly permeated the myometrium (Figure 2A,B). Several lymphovascular spaces were closely adjacent to the tumor cells, and a few foci of LVSI were identified. Both thin fibrovascular septa and dense hyalinized stroma surrounded the sheets and nests of tumor cells (Figure 2C,D). A solid, diffuse growth with little or no intervening stroma was also noted (Figure 2E). High-power magnification demonstrated a uniform population of large polygonal cells, possessing abundant clear or vacuolated cytoplasm and distinct cell borders. The tumor cells were mildly pleomorphic. Their round-to-ovoid nuclei were centrally or eccentrically located, with bland-looking chromatin and occasional punctate nucleoli. In some areas, eosinophilic intracytoplasmic materials were located near the tumor cell nuclei (Figure 2F). No severe nuclear pleomorphism, mitotic figure, or tumor cell necrosis were observed. Based on the morphological characteristics, we considered the possibility of perivascular epithelioid cell tumor (PEComa), clear cell sarcoma (CCS), metastatic renal cell carcinoma (RCC), granular cell tumor, paraganglioma, and ASPS [22], as these tumors share similar cytological features, such as abundant clear or eosinophilic granular cytoplasm with vacuoles.

#### 3.1.3. Results of Immunostaining and Special Staining

**Figure 3:**
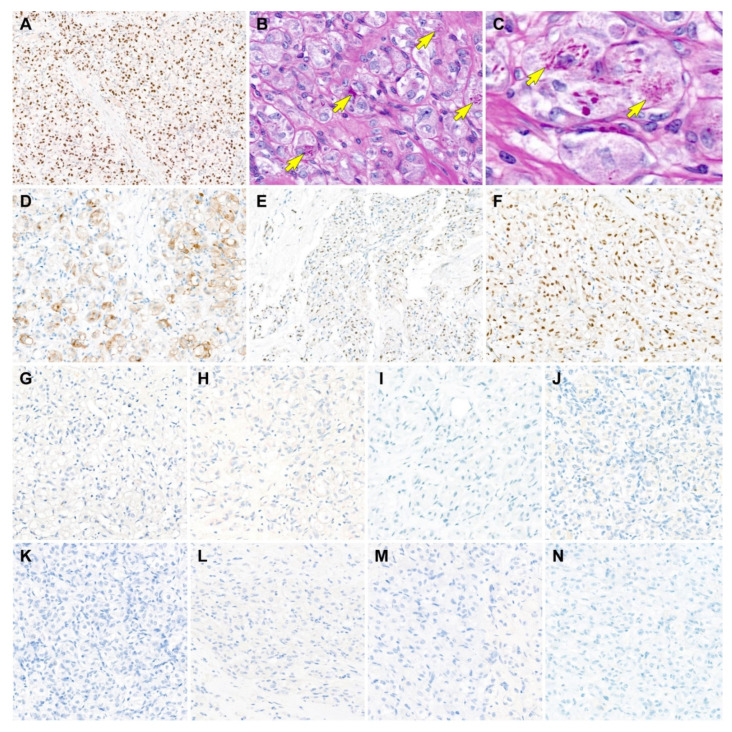
Results of immunostaining and special staining (case 1). (**A**) The tumor cells show uniform and strong nuclear immunoreactivity for transcription factor E3 (TFE3), a surrogate marker for ASPS chromosome region, candidate 1 (*ASPSCR1*)–*TFE3* fusion. (**B**,**C**) Periodic acid-Schiff with diastase digestion staining highlights the aggregates of eosinophilic intracytoplasmic rod-shaped crystalloids, which locate closely adjacent to the tumor cell nuclei (yellow arrows). (**D**–**F**) The tumor cells express (**D**) CD10, (**E**) estrogen receptor, and (**F**) progesterone receptor. (**G**–**M**) In contrast, the tumor cells are negative for (**G**) human melanoma black 45, (**H**) melan-A, (**I**) desmin, (**J**) S100, (**K**) pan-cytokeratin, (**L**) paired box 8, and (**M**) synaptophysin, excluding the possibility of *TFE3* translocation-associated perivascular epithelioid cell tumor, clear cell sarcoma, granular cell tumor, metastatic renal cell carcinoma, and paraganglioma. (**N**) MET expression is absent in the tumor cells. Original magnification: (**A**) 60×; (**B**) 400×; (**C**) 600×; (**D**) 200×; (**E**) 40×; (**F**–**N**) 200×.

All tumor cells showed uniform and strong nuclear immunoreactivity for transcription factor E3 (TFE3; staining percentage, 100%; Figure 3A), a surrogate marker for ASPS chromosome region, candidate 1 (*ASPSCR1*)–*TFE3* fusion [22,23]. PAS-D highlighted the aggregates of eosinophilic intracytoplasmic rod-shaped crystalloids (Figure 3B,C). The tumor cells also expressed CD10 (staining percentage, 70%; Figure 3D), estrogen receptor (ER; staining percentage, 30%; Figure 3E), and progesterone receptor (PR; staining percentage, 90%; Figure 3F), supporting the primary uterine origin [24]. On the other hand, the tumor cells were negative for human melanoma black 45 (HMB45; Figure 3G), melan-A (Figure 3H), desmin (Figure 3I), S100 (Figure 3J), pan-cytokeratin (pan-CK; Figure 3K), paired box 8 (PAX8; Figure 3L), and synaptophysin (Figure 3M), excluding the possibility of PEComa, CCS, metastatic RCC, granular cell tumor, and paraganglioma. MET immunostaining revealed a lack of MET-positive tumor cells (Figure 3N).

#### 3.1.4. NGS Results

NGS analysis revealed that the tumors harbored *ASPSCR1*–*TFE3* fusion. Lack of EWS RNA-binding protein 1 (*EWSR1*) translocation excluded CCS. No other pathogenic mutations or indels were detected.

### 3.2. Case 2: Primary ASPS of the Uterine Cervix

#### 3.2.1. Clinical Presentation

**Figure 4:**
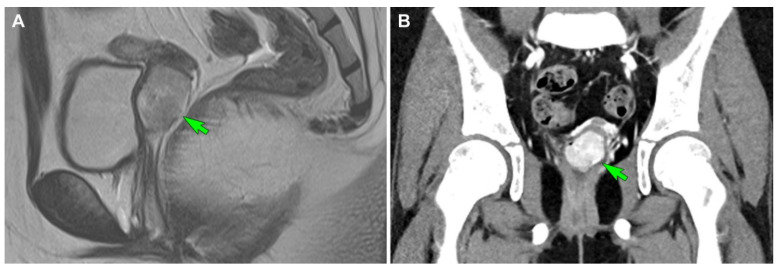
Imaging findings (case 2). (**A**) Initial T2-weighted turbo spin-echo sagittal magnetic resonance imaging reveals a well-circumscribed hypervascular mass (green arrow) originating in the uterine cervix, with heterogeneous high signal intensity and a lobulated contour. (**B**) Initial contrast-enhanced computed tomography shows an enhancing mass (green arrow) abutting the uterine cervix.

A 10-year-old girl presented with vaginal bleeding that lasted for more than a year. Physical examination revealed a 2.9 cm cervical mass. Pelvic MRI revealed a well-circumscribed hypervascular, lobulated mass in the uterine cervix (Figure 4A). Contrast-enhanced CT also showed an enhancing cervical mass (Figure 4B). After surgical excision of the mass, she was referred to our hospital for further management of ASPS. The imaging studies after mass excision could not completely exclude the possible presence of residual tumor in the cervix. MRI detected a small cervical lesion, which was suspected to be a residual tumor. The clinicians decided to perform a total hysterectomy after discussing with the patient and her parents.

#### 3.2.2. Pathological Findings

**Figure 5:**
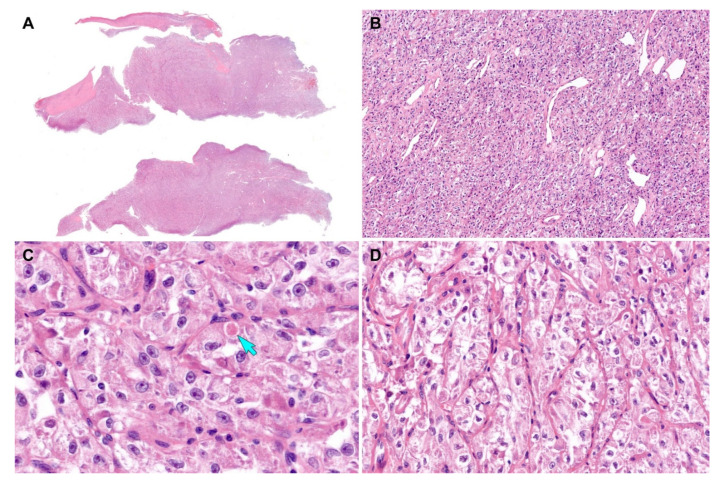
Histological findings (case 2). (**A**) Scanning-power magnification of the mass excision specimen reveals lobulated tumor tissue. (**B**) Low-power magnification reveals a diffuse growth pattern without nested architecture. Mildly dilated sinusoidal vascular channels are noted. (**C**) High-power magnification depicts eosinophilic intracytoplasmic globules (blue arrow) and pleomorphic nuclei with conspicuous nucleoli. (**D**) The discohesive tumor cells are arranged in a pseudoalveolar pattern in some areas. Original magnification: (**A**) 10×; (**B**) 40×; (**C**) 400×; (**D**) 200×.

We reviewed the outside pathology slides obtained from the mass excision specimen. Scanning-power magnification revealed a solid tumor with lobulated contour (Figure 5A). Low-power magnification showed a diffuse growth pattern without nested architecture or prominent vasculature (Figure 5B). The solid areas were highly cellular and consisted of relatively uniform tumor cells. Mildly dilated, sinusoid-like vascular channels were occasionally noted in-between solid cellular sheets. High-power magnification depicted eosinophilic intracytoplasmic globules and ample granular cytoplasm (Figure 5C). Most of the tumor cells had a centrally located, round nuclei showing mild pleomorphism and smooth nuclear membrane. Conspicuous nucleoli were rarely noted. In some areas, the discohesive tumor cells were arranged in a pseudoalveolar pattern (Figure 5D). No LVSI, tumor cell necrosis, or mitosis was detected. No residual tumor was observed in the hysterectomy specimen. A 0.8 cm small fibrotic lesion identified in the cervix exhibited post-surgical inflammation and fibrosis caused by the previous mass excision. Regarding the morphological features and the patient’s age, we considered ASPS to be the most probable diagnosis. Similar to the case 1, the differential diagnosis included PEComa, metastatic RCC, and paraganglioma. We excluded CCS and granular cell tumor based on the outside pathology report stating negative immunoreactivities for desmin and S100.

#### 3.2.3. Results of Special Staining and Immunostaining

**Figure 6:**
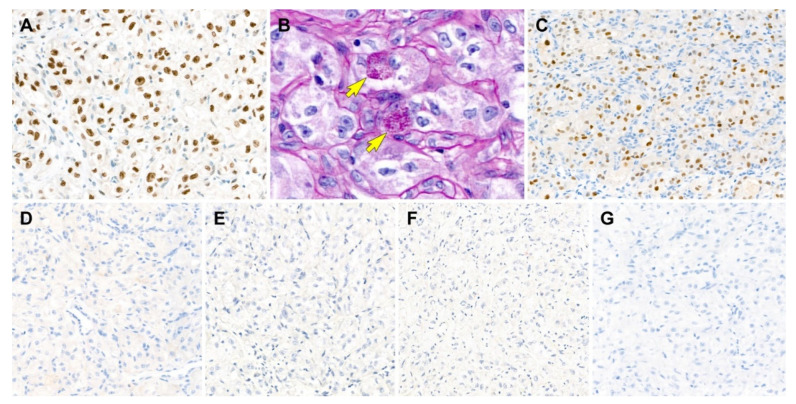
Results of immunostaining and special staining (case 2). (**A**) The tumor cells are diffusely positive for transcription factor E3 with moderate-to-strong staining intensity. (**B**) Periodic acid-Schiff with diastase digestion staining reveals eosinophilic granular materials within the cytoplasm (yellow arrows). (**C**) Similar to case 1, progesterone receptor is strongly positive for tumor cell nuclei. (**D**–**G**) The tumor cells are negative for (**D**) CD10, (**E**) human melanoma black 45, (**F**) melan-A, and (**G**) pan-cytokeratin. Original magnification: (**A**) 400×; (**B**) 600×; (**C**–**G**) 200×.

We performed PAS-D staining and immunostaining for HMB45, melan-A, pan-CK, CD10, and PR, using the outside unstained slides. The tumor cells were diffusely positive for TFE3 (staining percentage, 100%; Figure 6A) with moderate-to-strong staining intensity. PAS-D revealed eosinophilic granular materials within the cytoplasm (Figure 6B). PR was strongly positive in approximately 80% of the tumor cell nuclei (Figure 6C). In contrast, the tumor cells did not react with CD10 (Figure 6D), HMB45 (Figure 6E), melan-A (Figure 6F), and pan-CK (Figure 6G).

#### 3.2.4. NGS Results

NGS analysis revealed that the tumors harbored *ASPSCR1*–*TFE3* fusion. Lack of EWS RNA-binding protein 1 (*EWSR1*) translocation excluded CCS. No other pathogenic mutations or indels were detected.

### 3.3. Post-Operative Follow-Up

Both patients are currently well without evidence of disease recurrence or metastasis two (case 1) and four (case 2) months postoperatively.

## 4. Discussion

Table 2 summarizes the clinicopathological characteristics, immunophenotypes, and molecular alterations of our cases. We found some similarities and differences between the two tumors, each of them arose in the uterine corpus and cervix, respectively. Both patients presented with vaginal bleeding. Both tumors appeared as a polypoid mass, suggesting a favorable outcome as reported in the previous literature [25]. Both tumors displayed diffuse and strong nuclear PR immunoreactivity. In contrast, CD10, an endometrial stromal marker, was positive in case 1 only. This finding raises the possibility that ASPS of the uterine corpus might have a different cell of origin from that of the cervical primary. CD10 positivity in ASPS arising from the uterine corpus supports the hypothesis that it might originate from the endometrial stromal cells [24]. PAS-D staining revealed a morphological difference of intracytoplasmic materials between them. Case 1 showed relatively well-formed, rod-shaped crystalloids, whereas in case 2 coarse granular materials were identified within the cytoplasm.

The differential diagnosis of primary uterine ASPS includes PEComa, CCS, metastatic RCC, granular cell tumor, and paraganglioma. Differentiating ASPS from PEComa based just on morphology is sometimes challenging, as they share overlapping histological features, such as pseudoalveolar pattern and polygonal cells with clear or eosinophilic granular cytoplasm. Even though TFE3 is a surrogate marker for *ASPSCR1*–*TFE3* fusion, strong nuclear TFE3 immunoreactivity cannot exclude the possibility of *TFE3*-rearranged tumors including *TFE3* translocation-associated PEComa and Xp11.2 translocation RCC. Granular cell tumor can also strongly express TFE3 [25,26,27]. Therefore, additional immunostaining is mandatory to confirm the diagnosis of ASPS. Schoolmeester et al. [26] suggested HMB45, melan-A, and desmin as key markers of immunostaining panel to distinguish uterine conventional or *TFE3*-rearranged PEComa from ASPS. In this report, a lack of expression for all of these excluded PEComa. Absence of pan-CK and PAX8 immunoreactivity ruled out the possibility of metastatic RCC. S100 negativity excluded CCS and granular cell tumors [28].

To the best of our knowledge, 17 cases of primary uterine ASPS confirmed by either TFE3 immunostaining or molecular testing have been documented (Table 3) [25,26,27,28]. Among them, seven and eight cases were reported to arise in the uterine corpus and cervix, respectively. Two cases were reported to originate from the uterus, but a definite location was not documented. Uterine ASPS generally has a good prognosis, as only two patients developed pelvic lymph node metastasis [29] and postoperative recurrence [27], respectively. All except one patient had a small-sized (<5 cm) tumor, suggesting the association with a relatively favorable outcome compared to larger lesions in other locations [25]. However, a long-term follow-up of these cases is required to confirm the theory of uterine location being a site with favorable prognosis.

Recently, as the understanding of the molecular features of ASPS—a characteristic t(X;17)(p11;q25) and its correspondent chimeric *ASPSCR1*–*TFE3* fusion protein—has improved, attempts to develop novel drugs for ASPS are emerging [1]. Previous studies have reported that the production of *ASPSCR1*–*TFE3* fusion proteins leads to transcriptional upregulation and increased signaling of the MET pathway [26,35]. In a previous study by Jun et al. [36], MET expression was observed in six of eight cases of TFE3-positive ASPS. However, in this report, MET was completely negative in case 1. Large-scale case studies are necessary to clarify the potential therapeutic and prognostic significances of MET immunostaining and MET-targeted therapy.

## 5. Conclusions

We demonstrated the clinicopathological, immunophenotypical, and genetic features of two primary ASPS arising in the uterus. Primary uterine ASPS is an extremely rare malignant mesenchymal tumor, which has several morphological mimickers. Immunostaining with a panel of antibodies including TFE3 as well as markers for melanocytic, smooth muscle, neurogenic, and epithelial lesions is necessary for the differential diagnosis. The identification of *ASPSCR1*–*TFE3* fusion by molecular testing is helpful to confirm the diagnosis of ASPS. We believe our comprehensive and detailed analyses of primary uterine ASPS can help pathologists make an accurate histological diagnosis.

## Figures and Tables

**Table 1:** Antibodies used.

Antibody	Clone	Company	Dilution
CD10	56C6	Novocastra (Leica Biosystems, Buffalo Grove, IL, USA)	1:100
Pan-CK	AE1/AE3	Dako (Agilent Technologies, Santa Clara, CA, USA)	1:500
Desmin	D33	Dako (Agilent Technologies, Santa Clara, CA, USA)	1:200
ER	6F11	Novocastra (Leica Biosystems, Buffalo Grove, IL, USA)	1:300
PR	16	Novocastra (Leica Biosystems, Buffalo Grove, IL, USA)	1:1200
Ki-67	MIB1	Dako (Agilent Technologies, Santa Clara, CA, USA)	1:200
HMB-45	HMB45	Dako (Agilent Technologies, Santa Clara, CA, USA)	1:80
TFE3	MRQ-37	Cell Marque (Rocklin, CA, USA)	1:20
Melan-A	A103	Dako (Agilent Technologies, Santa Clara, CA, USA)	1:80
Synaptophysin	DAKO-SYNAP	Dako (Agilent Technologies, Santa Clara, CA, USA)	1:400
S100	Polyclonal	Dako (Agilent Technologies, Santa Clara, CA, USA)	1:5000
MET	SP44	Ventana (Roche, Darmstadt, Germany)	Prediluted

Abbreviations: ER, estrogen receptor; HMB45, human melanoma black 45; pan-CK, pan-cytokeratin; PR, progesterone receptor; TFE3, transcription factor E3.

**Table 2:** Summary of clinicopathological, immunophenotypical, and molecular characteristics of primary uterine alveolar soft part sarcoma.

Characteristic	Case 1	Case 2
Tumor location	Uterine corpus	Uterine cervix
Age at diagnosis	27 years	10 years
Presenting symptom	Vaginal bleeding	Vaginal bleeding
Initial clinical impression	Leiomyoma	Cervical mass
Primary treatment	Myomectomy	Mass excision
Definite treatment	TH, BS	TH
Hysterectomy diagnosis	2.5 cm residual ASPS	No residual ASPS
Post-operative treatment	None	None
Disease-free survival	2 months	4 months
Gross appearance	Polypoid mass	Polypoid mass
Histological growth pattern	Mainly solid	Mainly solid
Myometrial or cervical stromal involvement	Superficial myometrium	NA
PAS-D	Rod-shaped crystalloids	Coarse granular materials
TFE3 (staining %)	Positive (100)	Positive (100)
ER (staining %)	Positive (30)	NA
PR (staining %)	Positive (90)	Positive (80)
CD10 (staining %)	Positive (70)	Negative (0)
HMB45 (staining %)	Negative (0)	Negative (0)
Melan-A (staining %)	Negative (0)	Negative (0)
Desmin (staining %)	Negative (0)	Negative (0)
S100 (staining %)	Negative (0)	Negative (0)
Pan-CK (staining %)	Negative (0)	Negative(0)
PAX8 (staining %)	Negative (0)	NA
Synaptophysin (staining %)	Negative (0)	NA
MET (staining %)	Negative (0)	NA
*ASPSCR1*–*TFE3* fusion	Detected	Detected

Abbreviations: ASPS, alveolar soft part sarcoma; *ASPSCR1*–*TFE3*, ASPS chromosome region, candidate 1-transcription factor E3; BS, bilateral salpingectomy; ER, estrogen receptor; HMB45, human melanoma black 45; NA, not applicable; pan-CK, pan-cytokeratin; PAS-D, periodic acid-Schiff with diastase digestion; PAX8, paired box 8; PR, progesterone receptor; TFE3, transcription factor E3; TH, total hysterectomy.

**Table 3:** Summary of previously published cases of primary uterine alveolar soft part sarcoma.

No	Year Published	Authors	Age (Years)	Tumor Location	Tumor Size (cm)	Treatment	LN Metastasis	Follow-Up	DFS (Months)	Survival Status	Growth Pattern	TFE3 IHC	*ASPSCR1–TFE3* Fusion
1	2005	Roma et al. [30]	39	Cervix	0.2	TH	NA	NA	Recent	Alive	Nested	+	NA
2	2007	Kasahima et al. [24]	50	Corpus	1.9	TH, BSO, PLND	Absent	NED	38	Alive	Nested	+	NA
3	2011	Williams et al. [31]	31	Cervix	3.0	Excision	NA	NED	36	Alive	NA	+	Detected
4	2011	Hasegawa et al. [32]	56	Cervix	8.0	TH, BSO	NA	NED	66	Alive	Nested	+	Detected
5	2012	Zhang et al. [29]	57	Corpus	2.4	TH, BSO, PLND	Present	NED	9	Alive	Nested	+	NA
6	2014	Lee [33]	17	Cervix	1.6	Wide excision	NA	Recurred	40	Alive	Nested	+	NA
7	2014	Feng et al. [34]	21	Cervix	5.0	TH, BSO, PLND	Absent	NED	3	Alive	Nested	+	Detected
8	2017	Schoolmeester et al. [26]	37	Cervix	2.2	TH, BSO, PLND	Absent	NED	1	Alive	Nested	+	Detected
9	2017	Schoolmeester et al. [26]	45	Cervix	0.7	TH	NA	NA	Recent	Alive	Nested	+	Detected
10	2017	Schoolmeester et al. [26]	32	Corpus	NA	TH, BSO	NA	NED	8	Alive	Nested	+	Detected
11	2017	Schoolmeester et al. [26]	33	Corpus	NA	NA	NA	NA	NA	NA	Solid	+	Detected
12	2017	Schoolmeester et al. [26]	23	Corpus	NA	Curettage	NA	NED	9	Alive	Nested	+	Detected
13	2017	Schoolmeester et al. [26]	31	Uterus	NA	TH	NA	NED	35	Alive	Solid	+	Detected
14	2017	Schoolmeester et al. [26]	68	Uterus	NA	TH	NA	NED	15	Alive	Nested	+	Detected
15	2017	Zhang et al. [28]	68	Cervix	1.0	RH	NA	NA	NA	Alive	Nested	+	NA
16	2020	Gomez et al. [2]	20	Corpus	3.5	TH	NA	NED	NA	Alive	Nested	+	Detected
17	2021	Vishwajeet et al. [25]	24	Corpus	2.4	Mass excision	NA	NED	NA	Alive	Nested	+	NA

Abbreviations: *ASPSCR1*–*TFE3*, ASPS chromosome region, candidate 1-transcription factor E3; BSO, bilateral salpingo-oophorectomy; DFS, disease-free survival; LN, lymph node; NA, not applicable; NED, no evidence of disease; PLND, pelvic lymph node dissection; RH, radical hysterectomy; TFE3, transcription factor E3; TH, total hysterectomy.

## Data Availability

Not applicable.
